# One-Pot Hierarchical Structuring of Nanocellulose by Electrophoretic Deposition

**DOI:** 10.1021/acsnano.2c06392

**Published:** 2022-10-21

**Authors:** Takaaki Kasuga, Tsuguyuki Saito, Hirotaka Koga, Masaya Nogi

**Affiliations:** † SANKEN (The Institute of Scientific and Industrial Research), Osaka University, 8-1 Mihogaoka, Ibaraki, Osaka 567-0047, Japan; ‡ Department of Biomaterial Sciences, Graduate School of Agricultural and Life Sciences, The University of Tokyo, 1-1-1 Yayoi, Bunkyo-ku, Tokyo 113-8657, Japan

**Keywords:** nanocellulose, hydrogel, hierarchical structure, biomimicry, electrophoretic deposition, orientation control

## Abstract

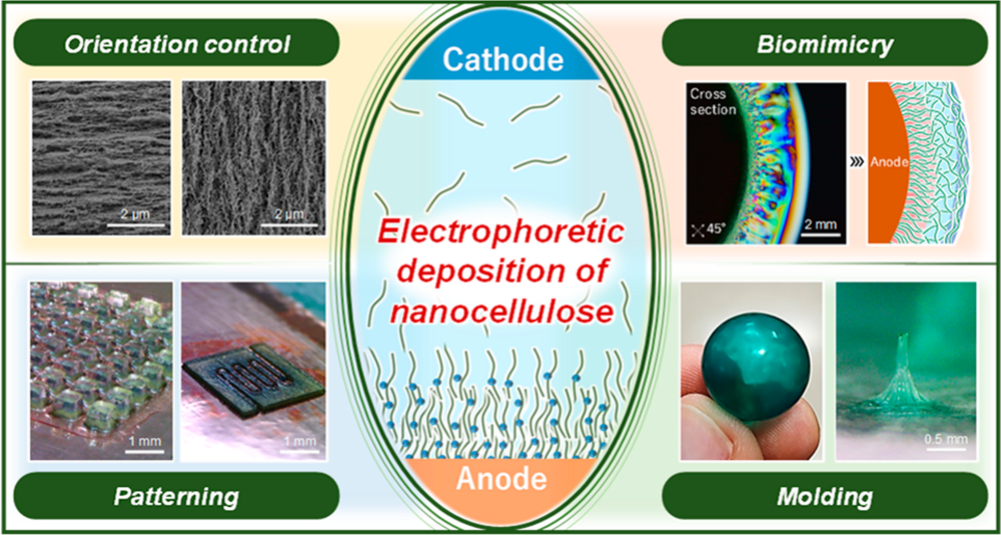

The orientation control and the formation of hierarchical structures of nanoscale components, such as bionanofibers and nanosheets, have attracted considerable research interest with the aim of achieving sophisticated functional materials. Herein, we report a simple and flexible strategy for constructing sophisticated hierarchical structures through electrophoretic and electrochemical deposition. Cellulose nanofibers (CNFs), which are used as model materials, are deposited on an anode in an aqueous dispersion and seamlessly oriented from horizontal to vertical relatively to the electrode by adjusting the applied voltage between the electrodes. The oriented CNF hydrogels not only exhibit anisotropic mechanical properties but also form complex orientations and hierarchical structures, such as cartilage- and plant stem-like configurations in response to electrode shape and applied voltage. This simple and flexible technique is expected to be applicable to various materials and contribute to a wide range of fields that include biomimicry, functional nanomaterials, and sustainable and functional moldings.

In nature, various living organisms use nanoscale components, such as nanofibers, to realize hierarchical structures that are highly strong and functional. Nanofibers, which are composed of biopolymers (e.g., cellulose, chitin, and collagen), form hierarchical structures with controlled orientations in living organisms to realize various functions necessary for the maintenance of life and the prosperity of the species.^1−5^ Plants, for example, contain cellulose nanofibers (CNFs) that are hierarchically oriented and form lightweight and robust structures.^1,2^ The anisotropic drying and shrinking phenomenon (“hygroscopic movement”) induced by the hierarchically oriented CNF structures contributes to species reproduction.^3,4^ Moreover, the cartilage in our bodies is known to contain collagen nanofibers that have highly oriented structures and provide both surface lubrication and cushioning.^1,5^ Hence, controlling the orientation and hierarchical structures of nanoscale components is critical for sustainability in living organisms.

The ability to structurally control nanomaterials has attracted considerable research interest with the aim of achieving particular functions, such as those of biological tissue.^6−21^ Previously, typical orientation control methods (e.g., shearing,^6,7^ stretching,^8,9^ applying alternating electric^10,11^ and magnetic fields,^12^ 3D printing,^13−17^ and directional freezing^18−20^) have been applied to aqueous dispersions of nanomaterials, including nanofibers and nanosheets. Among these, 3D printing is an effective method that enables orientation control and molding at the same time.^13−17^ Also, directional freezing has been attracting attention in recent years because of its ability to control orientation and structure on a multiscale.^18−20^ These methods have advantages in flexibility of application and have attracted attention from a wider range of fields than other orientation control methods. However, it is still difficult to simultaneously achieve multiaxial orientation control, multiscale structuring, and molding using a simple process.

In this regard, we developed an electrophoretic and electrochemical technique for the simple and flexible orientational and hierarchical structural control of nanoscale components. Recently, the electrophoretic deposition of CNFs,^22−24^ cellulose nanocrystals,^25,26^ and chitosan^27^ has been utilized as a coating or a film preparation method. However, existing reports have achieved random orientation or chiral nematic orientation via self-assembly. In this study, CNFs prepared by TEMPO oxidation, which are negatively charged in water, were used as model materials. CNFs were electrophoretically and electrochemically deposited on the anode surface in a horizontal, random, or vertical orientation that depended on the applied voltage. Orientation control was also effective for electrodes with complex geometries, with CNFs demonstrated to stack easily in differently orientated states. In addition, we demonstrated a wide range of application possibilities, including biomimicking hydrogels and CNF moldings.

## Results and Discussion

**Figure 1:**
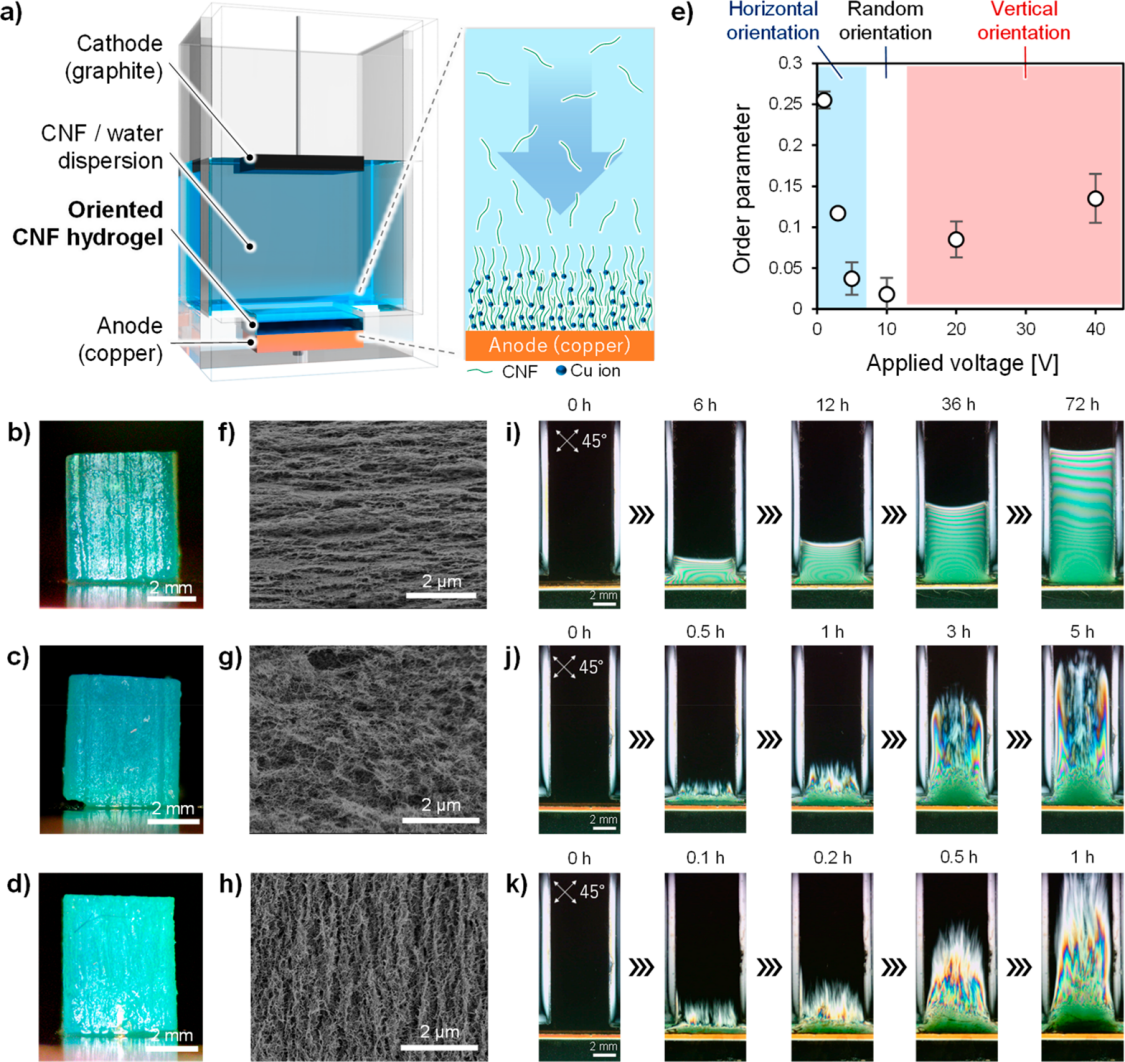
(a) Formation of oriented CNF hydrogel on the anode by sandwiching a CNF/water dispersion between the anode and cathode and applying a DC voltage between them. Oriented CNF hydrogels prepared at applied voltages of (b) 1, (c) 10, and (d) 40 V. CNF orientations at various applied voltages were investigated by (e) wide-angle X-ray diffractometry (WAXD), (f–h) field-emission scanning electron microscopy (FE-SEM), and (i–k) cross-polarizer-based techniques.

TEMPO-oxidized CNFs were used as model materials in this study. Copper and graphite electrodes were placed at the bottom and top of an acrylic cell containing a 0.2 wt % CNF/water dispersion, respectively (Figure 1a). CNFs were deposited on the anode at the bottom of the cell in hydrogel form when an applied voltage of 1–40 V (current density: 0.1–5 mA/cm^2^, Figure S1) was applied between the electrodes; CNFs were deposited on the anode at the bottom of the cell in a hydrogel form. The following actions contribute to the deposition of CNFs: electrophoresis by the electric field between the electrodes and two typical electrochemical reactions at the electrode/dispersion interface, elution of the electrode metal, and electrolysis of water.^22−24^ It has been reported that when a voltage exceeding the theoretical decomposition voltage of water (∼1.23 V) is applied, e.g., 3 V or more, the dispersion near the anode becomes acidic because of the electrolysis of water.^22−24^ The deposition rate of CNFs increased with increasing applied voltage (Figure 1i–k). The increase in the deposition rate might be attributable to the increase in the electric field strength and the elution rate of copper ions from the anode as well as acidification due to the electrolysis of water.^23,24^ The formed hydrogel appeared turquoise, which is ascribable to the adsorption of Cu ions by the carboxy groups of the CNFs (Figure 1b–d, Figures S2, S3). Interestingly, the CNFs were fixed in horizontal and vertical orientations with respect to the anode surface at applied voltages of 1 and 40 V, respectively (Figure 1e, f, h, i, k, Figure S4), while a close to randomly oriented state was observed at intermediate voltage (i.e., 10 V, Figure 1e, g, j); this state transformed into the horizontally or vertically oriented state at lower or higher applied voltage, respectively (Figure 1e).

Anisotropically oriented structures engender materials with anisotropic mechanical properties.^13^ The mechanical properties of the CNF hydrogels prepared using the electrophoretic and electrochemical method depend significantly on their orientation. The compressive moduli of the CNF hydrogels, for example, reflected their orientation states and were observed to be asymmetric with respect to the compression direction (Figure S5). The vertically oriented CNF hydrogel was less deformable under vertical compression and five times more deformable under horizontal compression. In addition, the surface of the horizontally oriented CNF hydrogel was observed to be smooth and have a low coefficient of friction compared to those of randomly oriented CNF hydrogels (Figure S6, Table S1).

**Figure 2:**
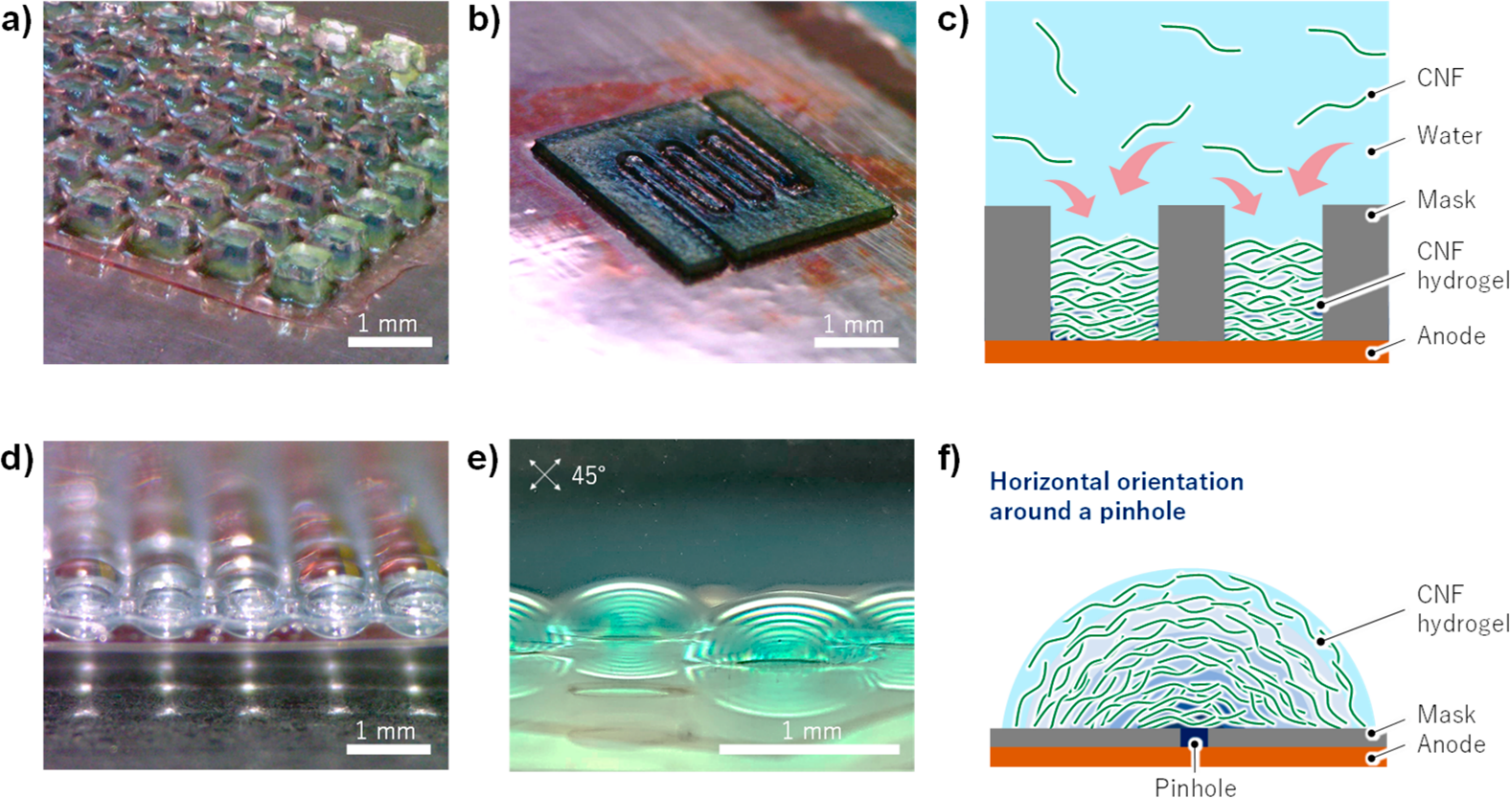
(a) Pillar arrays and (b) a flow channel were easily achieved. (c) Electrophoretic and electrochemical deposition of CNFs using a mask. (d) Hydrogel lens arrays fabricated using a pinhole mask. (e, f) The three-dimensional CNF oriented shape evaluated using a cross-polarizer.

The simplicity and flexibility of this method are the most important characteristic. We successfully created CNF hydrogels in the shapes of pillar arrays and flow channels by covering the anode with masks of various shape (Figure 2a, b, c). CNFs were deposited in concentric circles around the pinholes when a mask with pinholes was used, while hydrogel lens arrays were created when the horizontal orientation condition (applied voltage of 1 V) was applied (Figure 2d, e, f).

**Figure 3:**
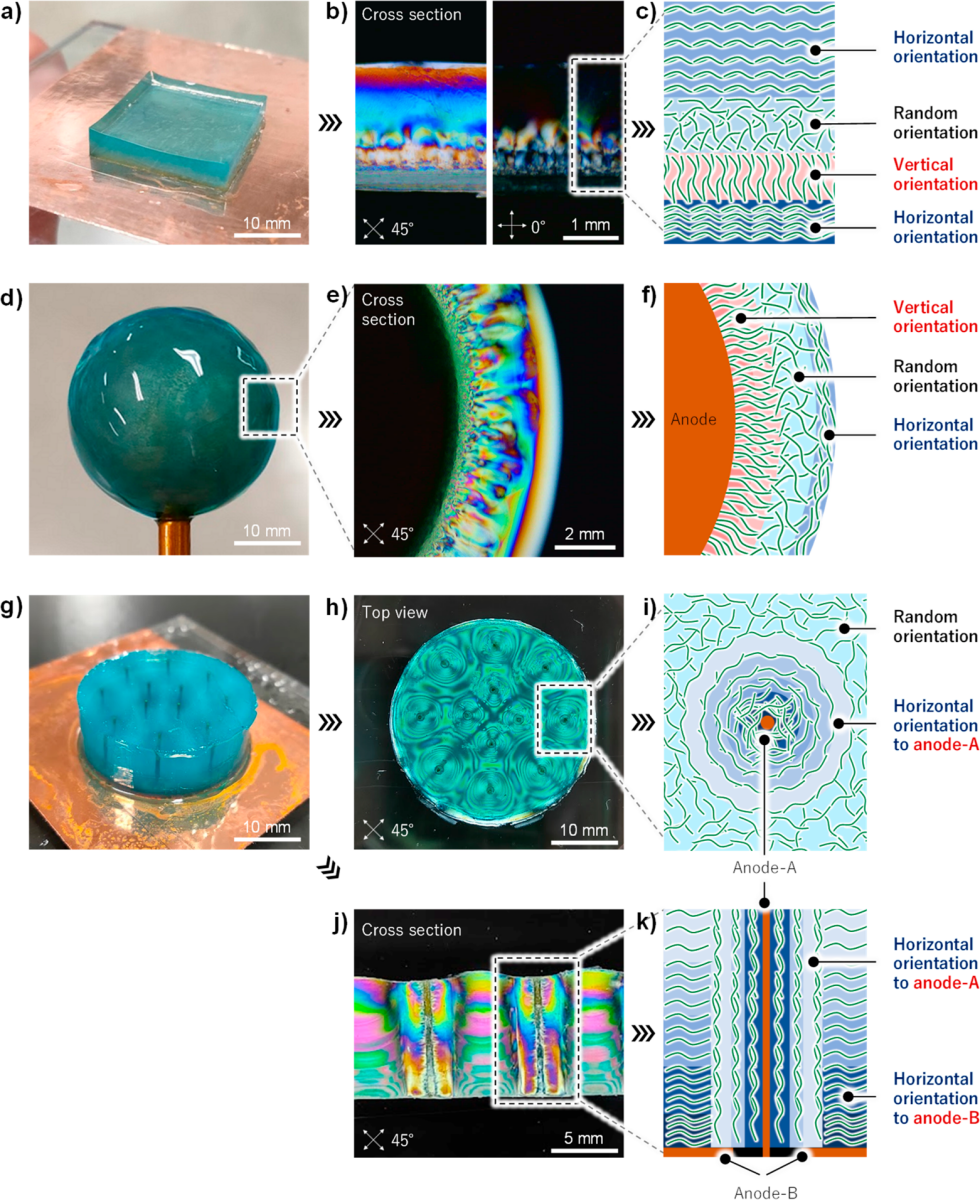
(a) Multilayered CNF hydrogel. (b) Cross-section of the multilayered CNF hydrogel observed through a cross-polarizer. (c) By controlling the applied voltage, CNFs were deposited in the order horizontal, vertical, random, and horizontal with respect to the anode. (d) Cartilage-like CNF hydrogel formed around a spherical electrode. (e) Cross-section observed through a cross-polarizer. (f) Corresponding schematic diagram. (g) Plant-stem-like CNF hydrogel prepared by combining wire and flat electrodes. (h, j) Corresponding cross-section. (i, k) Corresponding schematic diagram.

Multiscale and three-dimensional orientational control, such as in biological systems, has been achieved by the developed method. CNFs were deposited and fixed in any orientation state and at any thickness by tuning the applied voltage, even during the process. A multilayer hydrogel with continuously changing horizontal and vertical orientations was prepared by varying the applied voltage from 1 to 40 V and back to 1 V (Figure 3a–c). The electrode does not have to be flat; three-dimensional electrodes can also be used. A cartilage-like hydrogel capable of seamlessly transitioning from vertical to horizontal was prepared by inserting a spherical electrode in the CNF/water dispersion and varying the applied voltage from 40 to 1 V (Figure 3d–f). A three-dimensional plant-stem-like CNF hydrogel was prepared by combining a thin wire electrode with a plate electrode (Figure 3g–k).

**Figure 4:**
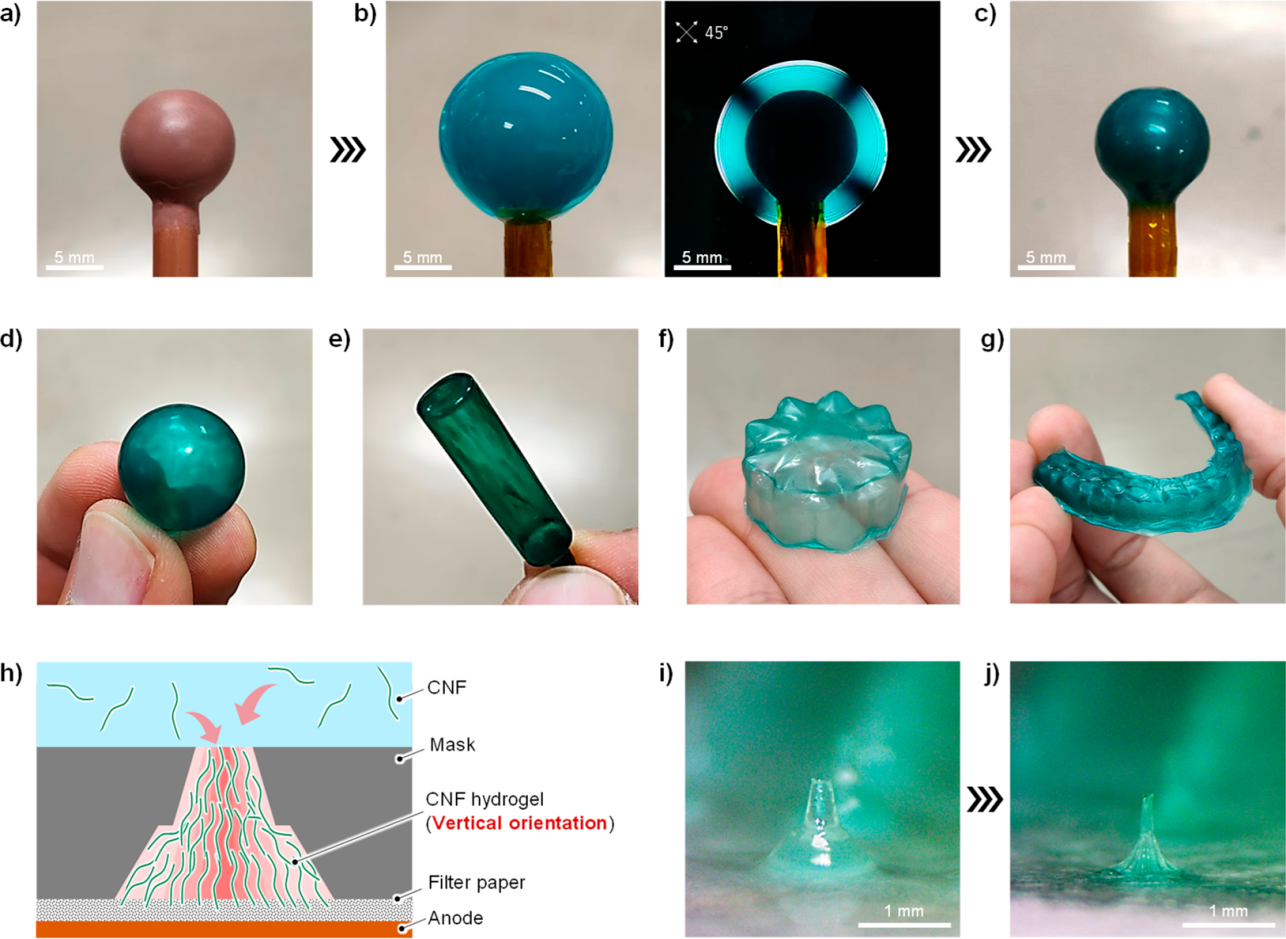
(a) Three-dimensional electrode, (b) horizontally oriented CNF hydrogel fixed on its surface, and (c, d) molded CNF film after drying the CNF hydrogel. Similarly obtained (e) cylindrical, (f) flower-like, and (g) mouthpiece-shaped molded CNF films. (h) Combining mask patterning, porous substrates, and vertically oriented CNFs. (i) CNF hydrogels with sharp structures fixed on porous substrates. (j) CNF microneedles after drying.

The applicability of the devised electrophoretic and electrochemical deposition method is not limited to the formation of oriented CNF hydrogels. The oriented CNF hydrogels anisotropically shrank upon drying, which is ascribable to the CNF orientation throughout the hydrogel (Figure S7). The anisotropic shrinking of the oriented CNF hydrogels was exploited for applications by combining it with three-dimensional orientation control and mask patterning. The CNFs formed in a horizontal orientation with respect to the electrode surface when deposited on a three-dimensional electrode at 1 V (Figure 4a, b). The subsequently formed horizontally oriented CNF hydrogel dried without cracking because drying-related shrinkage is suppressed in the horizontal direction, resulting in a smooth dry film on the electrode (Figure 4c). The electrodes were removed after drying to obtain molded CNF films with various three-dimensional shapes (Figure 4d–g). As for the vertical orientation, it was possible to form microneedle-like structures in combination with mask patterning by taking advantage of the fact that vertical shrinkage is suppressed during drying (Figure 4h–j). It is also possible to fix the CNF hydrogel and dried CNF material not only on the electrode surface but also on a porous material, such as filter paper. CNF moldings prepared by this method adsorb copper ions supplied from the anode. Therefore, in addition to the inherent properties of CNF structural materials (e.g., light weight, high strength, and high thermal resistance^28−32^), functional properties, such as antiviral properties toward COVID-19, were also provided (Table S2). In addition, the copper ions adsorbed on the CNF surface were easily removed by washing with hydrochloric acid^33^ (Figures S8, S9); therefore, it can be used flexibly according to the application. The introduced electrophoretic and electrochemical deposition method can be used to create a next generation of environmentally friendly alternatives to petroleum-based plastics.

CNF orientation can be controlled by several possible mechanisms in this study. Typical factors include the influence of the electric field and ion diffusion from the anode. There are several reports on the CNF orientation under the influence of an electric field.^10,11^ In most cases, an alternating-current electric field is used, which enables the CNFs to rotate vertically between the two electrodes in situ, which is ascribable to the anisotropic polarization properties of CNFs. However, such orientation requires a strong electric field of several hundred to several thousand V/cm. In this study, we used a direct-current electric field that is significantly weaker than those previously reported (∼12 V/cm). The effect of such a weak electric field on the CNF orientation state needs to be carefully evaluated.

Another possibility involves orientation through ion diffusion from the anode. Various polysaccharides,^34−36^ fibrous proteins,^37,38^ DNA,^39^ and semirigid polyanions^40^ have been reported to form anisotropic hydrogels through one-way ion diffusion. For example, sodium alginate is well known to orient horizontally with respect to the diffusion plane,^34^ while semirigid polyanions have been observed to orient horizontally or vertically with respect to the diffusion plane.^40^ In the electrophoretic and electrochemical method, copper ions, which are CNF cross-linkers (multivalent cations), are supplied from the anode, resulting in gelation. Gelation is caused by ion diffusion from one direction, and the orientation mechanism is likely to be the same as that previously reported. However, the CNF hydrogel becomes randomly^41^ or chirally nematically oriented through self-alignment^42^ when a CNF/water dispersion comes into contact with an acidic or aqueous multivalent cation solution from one direction to form a gel. This peculiar orientation phenomenon might be ascribable to the electrophoretic CNF concentration, which produces a concentration gradient suitable for orientation and hydrogel formation at the sol–gel interface.

**Figure 5:**
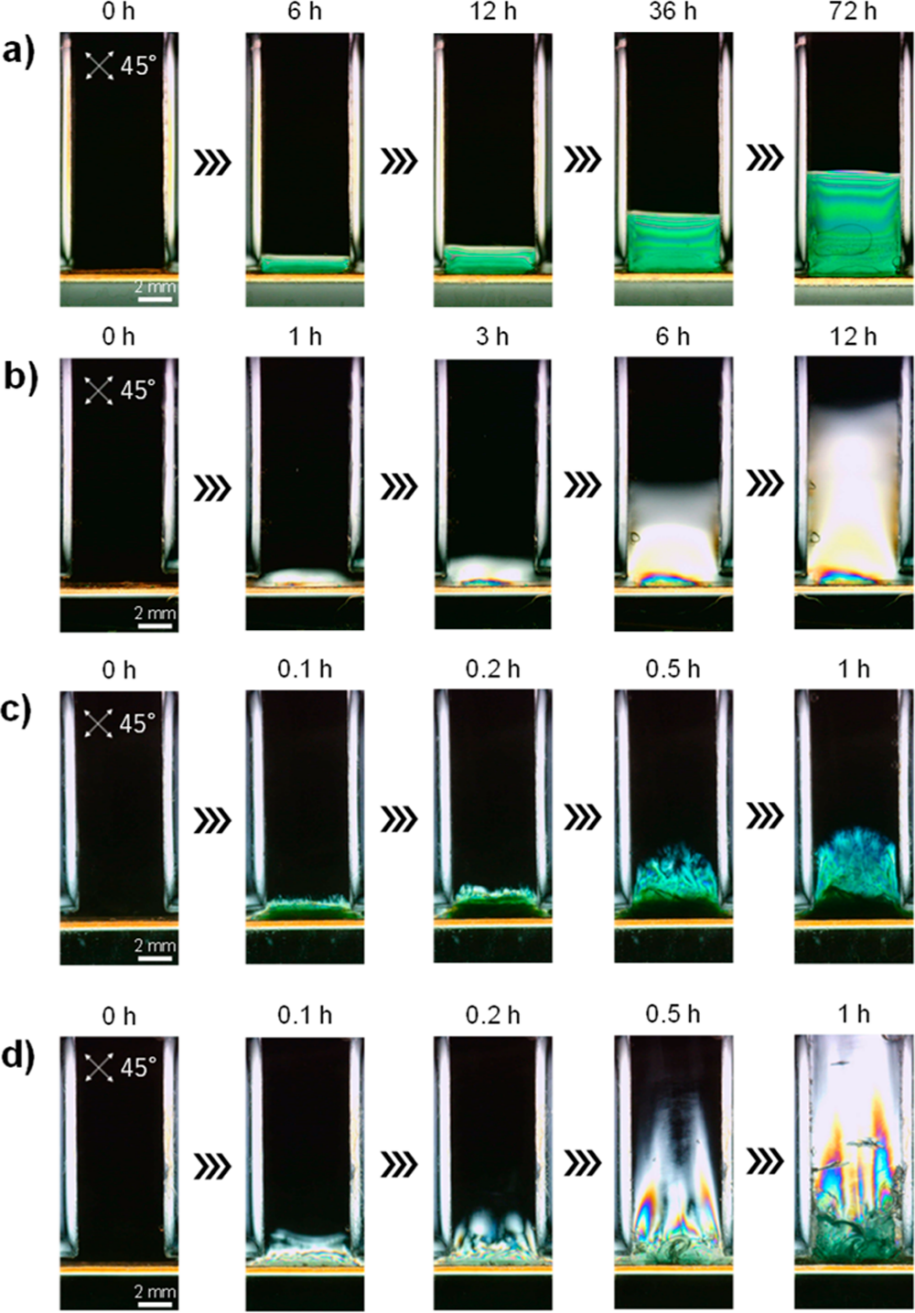
Horizontally oriented (a) sodium alginate hydrogel and (b) nanoclay hydrogel prepared at an applied voltage of 1 V observed through a cross-polarizer. Randomly and semivertically oriented (c) sodium alginate hydrogel and (d) nanoclay hydrogel prepared at an applied voltage of 40 V observed through a cross-polarizer.

The applicability of the method was tested on several anionic materials, including sodium alginate and nanoclay, which confirmed that the orientation depends significantly on the voltage conditions (Figures 5, S10). These results not only suggest that the method is applicable to a variety of materials but also realize highly structured nanocomposites and composites of two or more materials.

## Conclusion

A simple electrophoretic and electrochemical deposition method was developed for controlling and fixing CNF orientation, and its potential applications were investigated. CNFs were fixed in horizontal, random, and vertical orientations with respect to the anode surface by changing the applied voltage. Hierarchical structures, comprising CNFs with different layer-by-layer orientations, were formed in one-pot processes, forming complex geometries. Simplicity and flexibility are the strengths of this method. However, further research on the orientation mechanism is required. CNF size, concentration, dispersion temperature, voltage-application method (DC or pulsed), and the type of electrode metal may also affect the orientation state. Elucidating the mechanism responsible for electrophoretic- and electrochemical-deposition-induced orientational control and additional optimization is expected to assist in the development of nanomaterial composites that mimic biological tissue and the design of highly functional materials.

## Experimental Section

### Preparation of Cellulose Nanofiber Dispersions and Other Dispersions

TEMPO-oxidized cellulose pulp (carboxyl content, 1.8 mmol/g) was supplied by DKS Co., Ltd. The TEMPO-oxidized cellulose pulp slurry was homogenized using a high-pressure water-jet system (Star Burst, HJP-25008, Sugino Machine Co., Ltd., Japan) equipped with a ball-collision chamber. The injected slurry was repeatedly passed through a small nozzle (⦶ 0.15 mm) under a pressure of 200 MPa. A 0.2 wt % CNF/water dispersion was obtained after the slurry was passed through this nozzle 50 times. The width of the CNFs was ∼3 nm, and the average length of the CNFs was 181 ± 77 nm. The length of the CNFs was determined using an atomic force microscope (AFM, Nanocute, SII Nano Technology Inc., Japan) in the dynamic force microscope (DFM) mode and by using ImageJ software. Laboratory-grade sodium alginate was purchased from Nacalai Tesque, Inc. (Japan) and dissolved in distilled water at a concentration of 0.2 wt %. The nanoclay (laponite)/water dispersion was kindly provided by Dr. Zhifang Sun, RIKEN (Japan). Laponite XLG (LPN, thickness ∼1 nm; lateral size ∼25 nm) was purchased from Rockwood Additives Ltd. (UK). LPN powder (4.5 g) was dispersed in water (50 mL) and vigorously stirred for 10 min at 25 °C to form a homogeneous slurry with a concentration of 9 wt %. The slurry was diluted to 0.4 wt % before use.

### Electrophoretic and Electrochemical Deposition

Copper and graphite electrodes were placed at the bottom and top of an acrylic cell, respectively (anode size: 10 × 10 mm; distance between the electrodes: 35 mm). Voltage in the 1–40 V range was applied across the electrodes using a source measure unit (B2902A, Keysight Technologies, USA), and the cell was filled with a 0.2 wt % CNF/water dispersion (150 mL). The CNF/water dispersion was neutral (pH: ∼7), and the temperature was maintained at room temperature (∼25 °C) during the voltage application. An acrylic mask was placed on the electrode in certain contexts. A 0.2 wt % sodium alginate solution and a 0.4 wt % nanoclay dispersion were also used under the same conditions as the CNF/water dispersion. Cartilage-like CNF hydrogels were prepared by the electrophoretic deposition of CNFs on spherical copper electrodes, while the applied voltage was gradually decreased from 40 V to 1 V. Plant-stem-like CNF hydrogels were prepared by the deposition of CNFs at an applied voltage of 1 V using electrodes with fine wires arranged orthogonally over a flat electrode.

### Structural Characterization of CNF Hydrogels

A 15-mm-thick mask with a 10 × 10 mm square hole was used to create the CNF hydrogel. Voltages of 1, 10, and 40 V for 72, 10, and 2 h, respectively, were required to grow each hydrogel to a thickness of 15 mm. A 5 × 5 × 15 mm hollow was formed in the center of a 10 × 10 × 15 mm prismatic hydrogel and used to evaluate orientation. CNF aerogels were prepared by replacing ethanol in the CNF hydrogels, followed by supercritical drying (SYGLCP-8, Sanyu Gijutsu, Japan). Transmission X-ray diffractometry (XRD) was used to examine CNF orientation in the aerogels. The XRD patterns of the CNF aerogels were recorded on Fujifilm imaging plates under vacuum using a Rigaku MicroMax-007HF instrument operating at 40 kV and 30 mA with Cu Kα radiation (λ = 0.154 18 nm). The aerogels were set perpendicular to the X-ray beam, and the distances from the imaging plates were calibrated using NaF. The order parameter was calculated using a previously reported method.^6,8,9^ In this study, the order parameters range from 0 to 1, where 0 represents a random alignment and 1 represents perfect CNF alignment. The sample was placed at an angle of 0° or 45° to the cross-polarizer. The osmium-treated cross-sections of the CNF aerogels were examined by field-emission scanning electron microscopy (SEM; S-4800, Hitachi, Japan) at 1.5 kV.

### Preparation of CNF Moldings

Electrodes were prepared using copper-based conductive paint (SP-D-03, Freedom Custom Guitar Research Co. Ltd., Japan) and commercially available paraffin wax. CNFs were deposited on the meltable electrode surface in the horizontal orientation at an applied voltage of 1 V. The horizontally oriented CNF hydrogels formed on the electrode surface were dried at 10 °C and 90% RH. The molded CNF film was obtained through heating at 80 °C and washing with lacquer thinner to remove the electrodes. CNF microneedles were prepared by drying vertically oriented CNF hydrogels patterned with a mask at an applied voltage of 40 V on filter paper, at 10 °C and 90% RH.

## Supporting Information Available

Typical current density changes during voltage application, FT-IR and XRF spectra of CNF aerogels, WAXS images and azimuthal intensity profiles for CNF aerogels, compressive moduli of CNF hydrogels in different orientations, the demonstration of scalability of CNF hydrogel formation, drying-related shrinkage of CNF hydrogels, removal of copper ions adsorbed on CNFs by hydrochloric acid, cross-section of an alginate hydrogel and a nanoclay hydrogel, results of friction coefficient measurements for CNF hydrogels, and antiviral testing of molded CNF films against SARS-CoV-2 (PDF)

## References

[ref1] Fratzl P.; Weinkamer R. Nature’s Hierarchical Materials. Prog. Mater. Sci. 2007, 52 (8), 1263–1334. 10.1016/j.pmatsci.2007.06.001.

[ref2] Gibson L. J. The Hierarchical Structure and Mechanics of Plant Materials. J. R. Soc. Interface. 2012, 9 (76), 2749–2766. 10.1098/rsif.2012.0341. 22874093 PMC3479918

[ref3] Allen R.; Wardrop A. The Opening and Shedding Mechanism of the Female Cones of Pinus Radiata. Aust. J. Bot. 1964, 12 (2), 125 10.1071/BT9640125.

[ref4] Elbaum R.; Gorb S.; Fratzl P. Structures in the Cell Wall That Enable Hygroscopic Movement of Wheat Awns. J. Struct. Biol. 2008, 164 (1), 101–107. 10.1016/j.jsb.2008.06.008. 18625323

[ref5] Sophia Fox A. J.; Bedi A.; Rodeo S. A. The Basic Science of Articular Cartilage: Structure, Composition, and Function. Sports Health 2009, 1 (6), 461–468. 10.1177/1941738109350438. 23015907 PMC3445147

[ref6] Lin X. Y.; Wang Z. J.; Pan P.; Wu Z. L.; Zheng Q. Monodomain Hydrogels Prepared by Shear-Induced Orientation and Subsequent Gelation. RSC Adv. 2016, 6 (97), 95239–95245. 10.1039/C6RA17103F.

[ref7] Yoshiharu N.; Shigenori K.; Masahisa W.; Takeshi O. Cellulose Microcrystal Film of High Uniaxial Orientation. Macromolecules 1997, 30 (20), 6395–6397. 10.1021/ma970503y.

[ref8] Choi S.; Kim J. Designed Fabrication of Super-Stiff, Anisotropic Hybrid Hydrogels via Linear Remodeling of Polymer Networks and Subsequent Crosslinking. J. Mater. Chem. B 2015, 3 (8), 1479–1483. 10.1039/C4TB01852D. 32262420

[ref9] Uetani K.; Okada T.; Oyama H. T. In-Plane Anisotropic Thermally Conductive Nanopapers by Drawing Bacterial Cellulose Hydrogels. ACS Macro Lett. 2017, 6 (4), 345–349. 10.1021/acsmacrolett.7b00087. 35610858

[ref10] Wise H. G.; Takana H.; Ohuchi F.; Dichiara A. B. Field-Assisted Alignment of Cellulose Nanofibrils in a Continuous Flow-Focusing System. ACS Appl. Mater. Interfaces 2020, 12 (25), 28568–28575. 10.1021/acsami.0c07272. 32453552

[ref11] Brouzet C.; Mittal N.; Rosén T.; Takeda Y.; Söderberg L. D.; Lundell F.; Takana H. Effect of Electric Field on the Hydrodynamic Assembly of Polydisperse and Entangled Fibrillar Suspensions. Langmuir 2021, 37 (27), 8339–8347. 10.1021/acs.langmuir.1c01196. 34176263

[ref12] Kimura F.; Kimura T.; Tamura M.; Hirai A.; Ikuno M.; Horii F. Magnetic Alignment of the Chiral Nematic Phase of a Cellulose Microfibril Suspension. Langmuir 2005, 21 (5), 2034–2037. 10.1021/la0475728. 15723507

[ref13] Markstedt K.; Mantas A.; Tournier I.; Martínez Ávila H.; Hägg D.; Gatenholm P. 3D Bioprinting Human Chondrocytes with Nanocellulose–Alginate Bioink for Cartilage Tissue Engineering Applications. Biomacromolecules 2015, 16 (5), 1489–1496. 10.1021/acs.biomac.5b00188. 25806996

[ref14] Håkansson K. M. O.; Fall A. B.; Lundell F.; Yu S.; Krywka C.; Roth S. V.; Santoro G.; Kvick M.; Prahl Wittberg L.; Wågberg L.; Söderberg L. D. Hydrodynamic Alignment and Assembly of Nanofibrils Resulting in Strong Cellulose Filaments. Nat. Commun. 2014, 5 (1), 4018 10.1038/ncomms5018. 24887005 PMC4059937

[ref15] Uetani K.; Koga H.; Nogi M. Checkered Films of Multiaxis Oriented Nanocelluloses by Liquid-Phase Three-Dimensional Patterning. Nanomaterials 2020, 10 (5), 958 10.3390/nano10050958. 32443531 PMC7281742

[ref16] Kilian D.; Ahlfeld T.; Akkineni A. R.; Bernhardt A.; Gelinsky M.; Lode A. 3D Bioprinting of Osteochondral Tissue Substitutes – in Vitro-Chondrogenesis in Multi-Layered Mineralized Constructs. Sci. Rep. 2020, 10 (1), 8277 10.1038/s41598-020-65050-9. 32427838 PMC7237416

[ref17] Saadi M. A. S. R.; Maguire A.; Pottackal N. T.; Thakur M. S. H.; Ikram M. Md.; Hart A. J.; Ajayan P. M.; Rahman M. M. Direct Ink Writing: A 3D Printing Technology for Diverse Materials. Adv. Mater. 2022, 34, 2108855 10.1002/adma.202108855. 35246886

[ref18] Chen Y.; Li S.; Li X.; Mei C.; Zheng J.; E S.; Duan G.; Liu K.; Jiang S. Liquid Transport and Real-Time Dye Purification via Lotus Petiole-Inspired Long-Range-Ordered Anisotropic Cellulose Nanofibril Aerogels. ACS Nano 2021, 15 (12), 20666–20677. 10.1021/acsnano.1c10093. 34881863

[ref19] Lin Z.; Dong J.; Wang X.; Huang Q.; Shen X.; Yang M.; Sun X.; Yuan Y.; Wang S.; Ning Y.; Yang S.; Yin W.; Li M.; Sun Y.; Zhang Q.; Li Y. Twin-Structured Graphene Metamaterials with Anomalous Mechanical Properties. Adv. Mater. 2022, 34 (17), 2200444 10.1002/adma.202200444. 35218071

[ref20] Shahbazi M.-A.; Ghalkhani M.; Maleki H. Directional Freeze-Casting: A Bioinspired Method to Assemble Multifunctional Aligned Porous Structures for Advanced Applications. Adv. Eng. Mater. 2020, 22 (7), 2000033 10.1002/adem.202000033.

[ref21] Sano K.; Ishida Y.; Aida T. Synthesis of Anisotropic Hydrogels and Their Applications. Angew. Chem., Int. Ed. 2018, 57 (10), 2532–2543. 10.1002/anie.201708196. 29034553

[ref22] Kasuga T.; Yagyu H.; Uetani K.; Koga H.; Nogi M. Cellulose Nanofiber Coatings on Cu Electrodes for Cohesive Protection against Water-Induced Short-Circuit Failures. ACS Appl. Nano Mater. 2021, 4 (4), 3861–3868. 10.1021/acsanm.1c00267.

[ref23] Kim H.; Endrődi B.; Salazar-Alvarez G.; Cornell A. One-Step Electro-Precipitation of Nanocellulose Hydrogels on Conducting Substrates and Its Possible Applications: Coatings, Composites, and Energy Devices. ACS Sustainable Chem. Eng. 2019, 7 (24), 19415–19425. 10.1021/acssuschemeng.9b04171.

[ref24] Guo X.; Gao H.; Zhang J.; Zhang L.; Shi X.; Du Y. One-Step Electrochemically Induced Counterion Exchange to Construct Free-Standing Carboxylated Cellulose Nanofiber/Metal Composite Hydrogels. Carbohydr. Polym. 2021, 254, 117464 10.1016/j.carbpol.2020.117464. 33357923

[ref25] Chen Q.; de Larraya U. P.; Garmendia N.; Lasheras-Zubiate M.; Cordero-Arias L.; Virtanen S.; Boccaccini A. R. Electrophoretic Deposition of Cellulose Nanocrystals (CNs) and CNs/Alginate Nanocomposite Coatings and Free Standing Membranes. Colloids Surfaces B Biointerfaces 2014, 118, 41–48. 10.1016/j.colsurfb.2014.03.022. 24727117

[ref26] Atifi S.; Mirvakili M.-N.; Williams C. A.; Bay M. M.; Vignolini S.; Hamad W. Y.; Atifi S.; Mirvakili M.-N.; Hamad W. Y.; Williams C. A.; Bay M. M.; Vignolini S. Fast Self-Assembly of Scalable Photonic Cellulose Nanocrystals and Hybrid Films via Electrophoresis. Adv. Mater. 2022, 34 (12), 2109170 10.1002/ADMA.202109170. 35076132

[ref27] Avcu E.; Baştan F. E.; Abdullah H. Z.; Rehman M. A. U.; Avcu Y. Y.; Boccaccini A. R. Electrophoretic Deposition of Chitosan-Based Composite Coatings for Biomedical Applications: A Review. Prog. Mater. Sci. 2019, 103, 69–108. 10.1016/j.pmatsci.2019.01.001.

[ref28] Nogi M.; Iwamoto S.; Nakagaito A. N.; Yano H. Optically Transparent Nanofiber Paper. Adv. Mater. 2009, 21 (16), 1595–1598. 10.1002/adma.200803174.

[ref29] Fukuzumi H.; Saito T.; Iwata T.; Kumamoto Y.; Isogai A. Transparent and High Gas Barrier Films of Cellulose Nanofibers Prepared by TEMPO-Mediated Oxidation. Biomacromolecules 2009, 10 (1), 162–165. 10.1021/bm801065u. 19055320

[ref30] Yagyu H.; Ifuku S.; Nogi M. Acetylation of Optically Transparent Cellulose Nanopaper for High Thermal and Moisture Resistance in a Flexible Device Substrate. Flex. Print. Electron. 2017, 2 (1), 014003 10.1088/2058-8585/aa60f4.

[ref31] Kasuga T.; Isobe N.; Yagyu H.; Koga H.; Nogi M. Clearly Transparent Nanopaper from Highly Concentrated Cellulose Nanofiber Dispersion Using Dilution and Sonication. Nanomaterials 2018, 8 (2), 104 10.3390/nano8020104. 29439544 PMC5853735

[ref32] Kasuga T.; Yagyu H.; Uetani K.; Koga H.; Nogi M. Return to the Soil” Nanopaper Sensor Device for Hyperdense Sensor Networks. ACS Appl. Mater. Interfaces 2019, 11 (46), 43488–43493. 10.1021/acsami.9b13886. 31659891

[ref33] Isobe N.; Chen X.; Kim U. J.; Kimura S.; Wada M.; Saito T.; Isogai A. TEMPO-Oxidized Cellulose Hydrogel as a High-Capacity and Reusable Heavy Metal Ion Adsorbent. J. Hazard. Mater. 2013, 260, 195–201. 10.1016/j.jhazmat.2013.05.024. 23747479

[ref34] Narita T.; Tokita M. Liesegang Pattern Formation in κ-Carrageenan Gel. Langmuir 2006, 22 (1), 349–352. 10.1021/la0522350. 16378443

[ref35] Dobashi T.; Tomita N.; Maki Y.; Chang C. P.; Yamamoto T. An Analysis of Anisotropic Gel Forming Process of Chitosan. Carbohydr. Polym. 2011, 84 (2), 709–712. 10.1016/j.carbpol.2010.07.004.

[ref36] Maki Y.; Ito K.; Hosoya N.; Yoneyama C.; Furusawa K.; Yamamoto T.; Dobashi T.; Sugimoto Y.; Wakabayashi K. Anisotropic Structure of Calcium-Induced Alginate Gels by Optical and Small-Angle X-Ray Scattering Measurements. Biomacromolecules 2011, 12 (6), 2145–2152. 10.1021/bm200223p. 21504159

[ref37] Furusawa K.; Sato S.; Masumoto J.; Hanazaki Y.; Maki Y.; Dobashi T.; Yamamoto T.; Fukui A.; Sasaki N. Studies on the Formation Mechanism and the Structure of the Anisotropic Collagen Gel Prepared by Dialysis-Induced Anisotropic Gelation. Biomacromolecules 2012, 13 (1), 29–39. 10.1021/bm200869p. 22107030

[ref38] Mredha Md. T. I.; Zhang X.; Nonoyama T.; Nakajima T.; Kurokawa T.; Takagi Y.; Gong J. P. Swim Bladder Collagen Forms Hydrogel with Macroscopic Superstructure by Diffusion Induced Fast Gelation. J. Mater. Chem. B 2015, 3 (39), 7658–7666. 10.1039/C5TB00877H. 32264576

[ref39] Furusawa K.; Minamisawa Y.; Dobashi T.; Yamamoto T. Dynamics of Liquid Crystalline Gelation of DNA. J. Phys. Chem. B 2007, 111 (51), 14423–14430. 10.1021/jp076135. 18047316

[ref40] Wu Z. L.; Kurokawa T.; Sawada D.; Hu J.; Furukawa H.; Gong J. P. Anisotropic Hydrogel from Complexation-Driven Reorientation of Semirigid Polyanion at Ca ^2+^ Diffusion Flux Front. Macromolecules 2011, 44 (9), 3535–3541. 10.1021/ma2001228.

[ref41] Dong H.; Snyder J. F.; Williams K. S.; Andzelm J. W. Cation-Induced Hydrogels of Cellulose Nanofibrils with Tunable Moduli. Biomacromolecules 2013, 14 (9), 3338–3345. 10.1021/bm400993f. 23919541

[ref42] Saito T.; Uematsu T.; Kimura S.; Enomae T.; Isogai A. Self-Aligned Integration of Native Cellulose Nanofibrils towards Producing Diverse Bulk Materials. Soft Matter 2011, 7 (19), 8804 10.1039/c1sm06050c.

