# Supplementary material for: One-Pot Hierarchical Structuring of Nanocellulose by Electrophoretic Deposition
Source: ACS Nano. 2022 Oct 21;16(11):18390–7. doi: 10.1021/acsnano.2c06392 (PMC9706670; doi:10.1021/acsnano.2c06392)
Supplement: Supplementary file 1 — nn2c06392_si_001.pdf [file nn2c06392_si_001.pdf]

Supporting Information

One-pot hierarchical structuring of nanocellulose by electrophoretic deposition

Takaaki Kasuga[1,*], *Tsuguyuki Saito*[2], *Hirotaka Koga*[1], *Masaya Nogi*[1]

[1] SANKEN (The Institute of Scientific and Industrial Research), Osaka University, 8-1 Mihogaoka, Ibaraki, Osaka 567-0047, Japan

E-mail: tkasuga@eco.sanken.osaka-u.ac.jp

[2] Department of Biomaterial Sciences, Graduate School of Agricultural and Life Sciences, The University of Tokyo, 1-1-1 Yayoi, Bunkyo-ku, Tokyo 113-8657, Japan

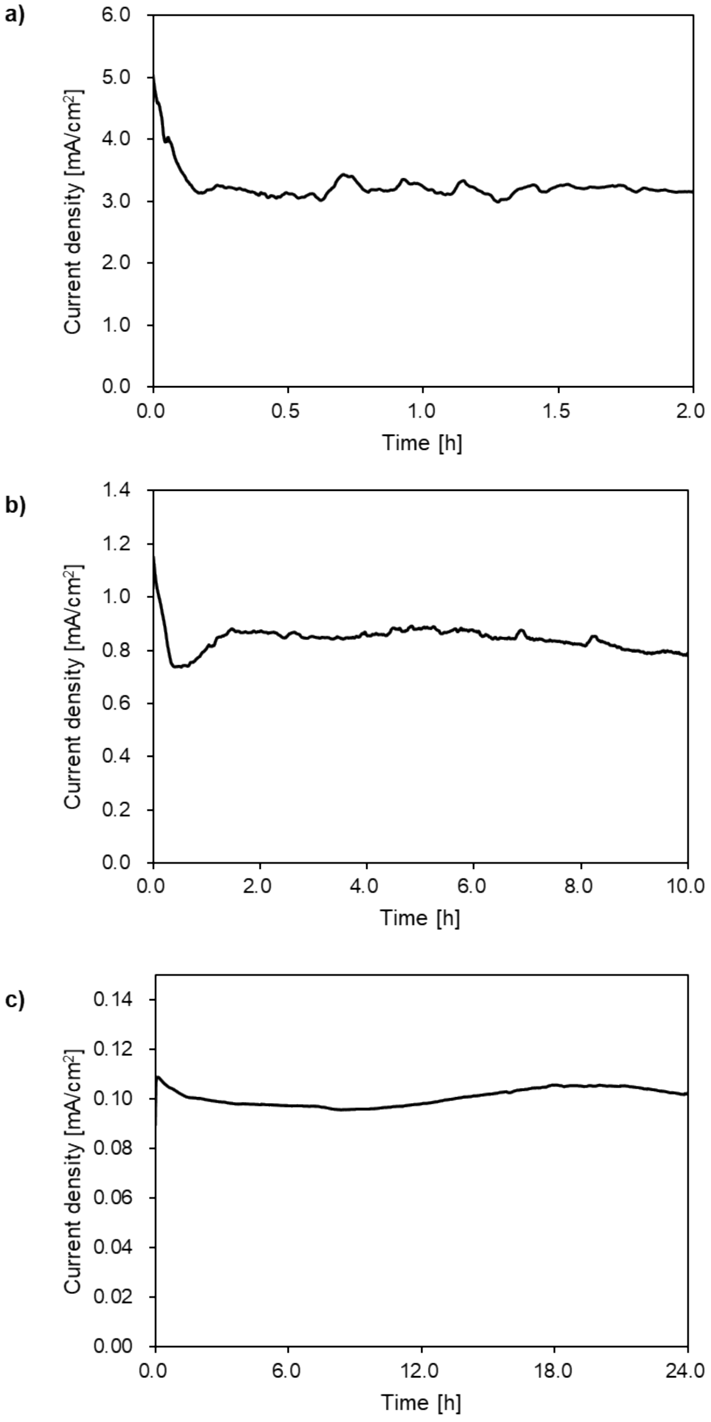

Figure S1. Typical current density changes when (a) 1 V, (b) 10 V, and (c) 40 V were applied.

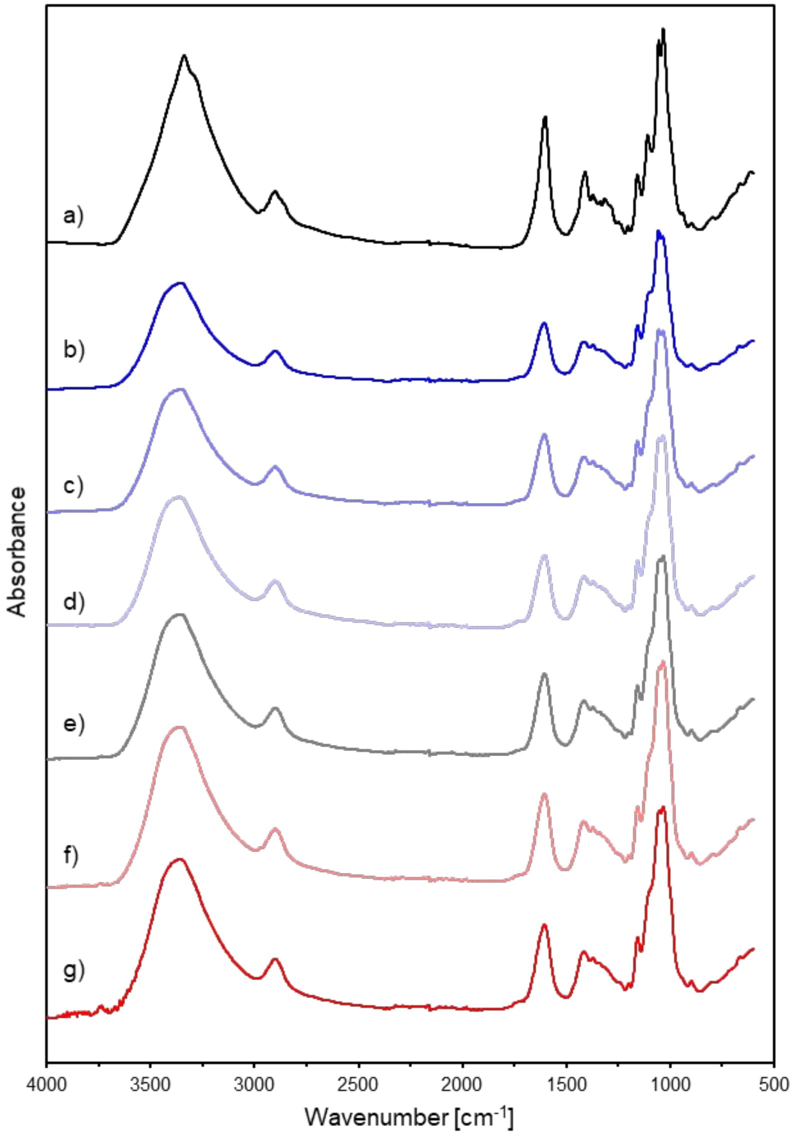

Figure S2. FT-IR spectra of (a) an original CNF and CNF aerogels prepared at applied voltage of (b) 1 V, (c) 3 V, (d) 5 V, (e) 10 V, (f) 20 V, (g) 40 V.

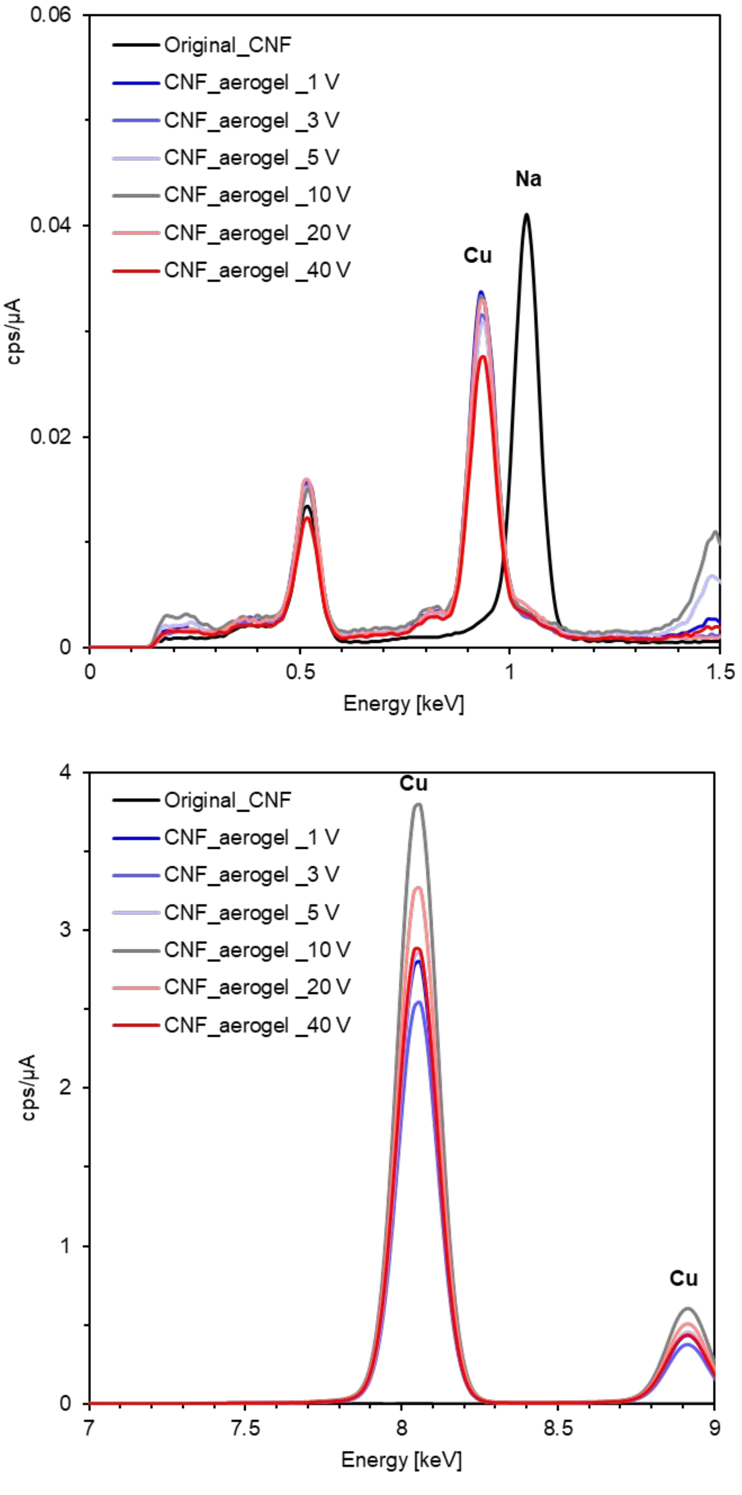

Figure S3. XRF spectra of the original CNF pulp and CNF aerogels prepared at applied voltages of 1–40 V.

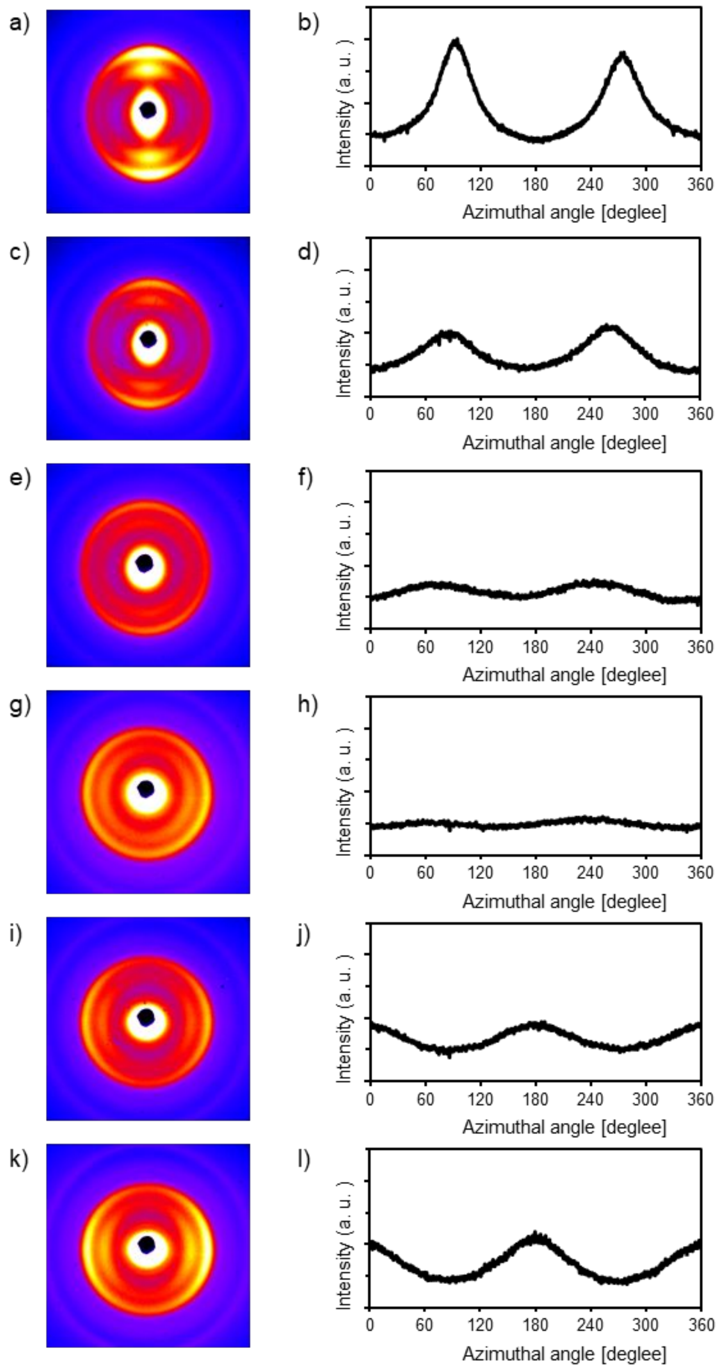

Figure S4. WAXS images and azimuthal intensity profiles at 2θ = 22.8° [(200) reflection of the cellulose I crystals] for CNF aerogels prepared at an applied voltage of (a, b) 1 V, (c, d) 3 V, (e, f) 5 V, **(**g, h) 10 V, (i, j) 20 V, and (k, l) 40 V.

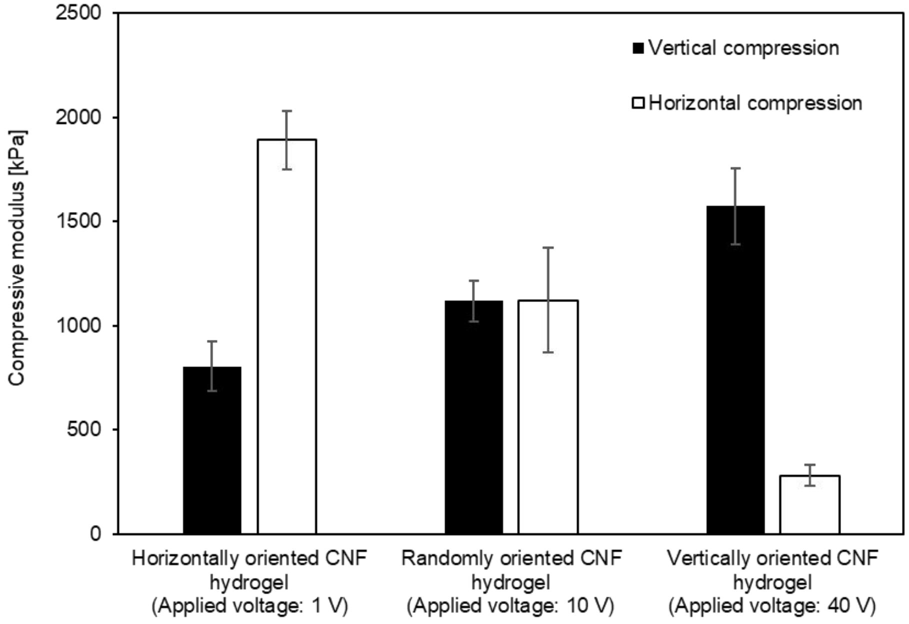

Figure S5. Compressive moduli of CNF hydrogels in different orientations obtained by horizontal and vertical compression tests (CNF concentration in each hydrogel: 8–9 wt%).

a)

b)

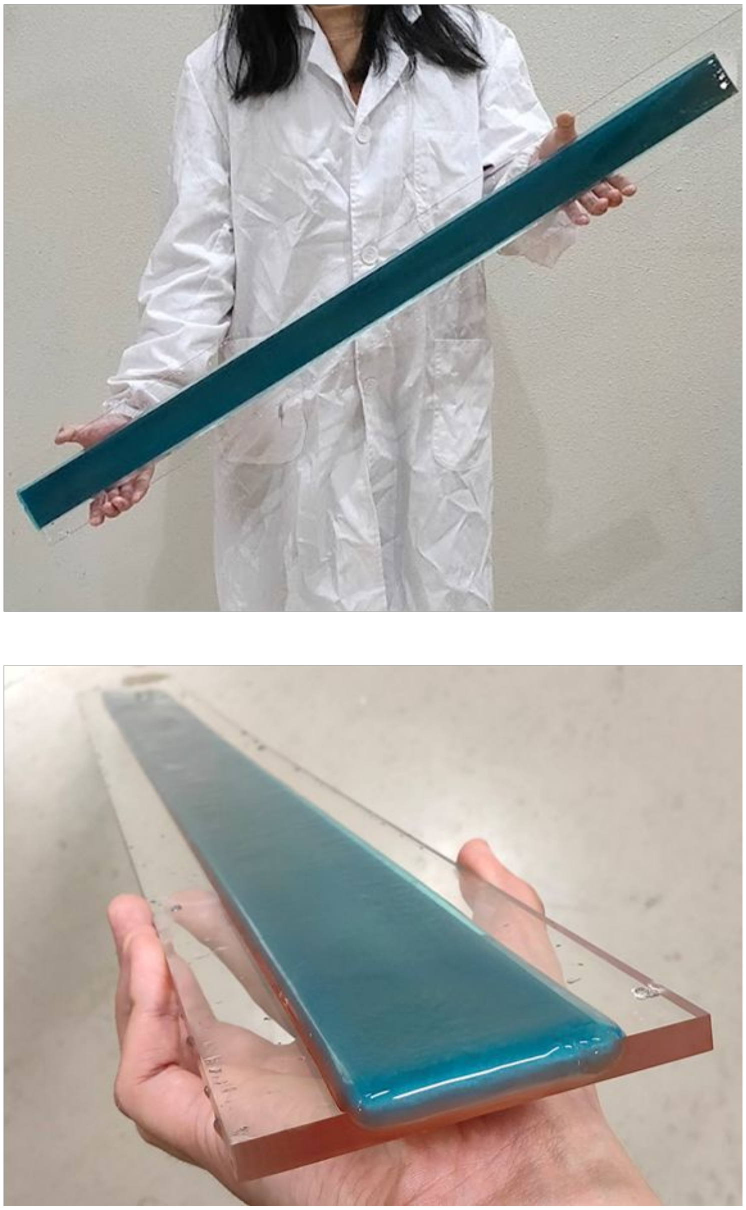

Figure S6. (a) Electrophoretic and electrochemical deposition method was applicable to large areas. (b) When CNF was deposited under horizontally oriented conditions (applied voltage: 1 V), the electrode was covered with a smooth CNF hydrogel.

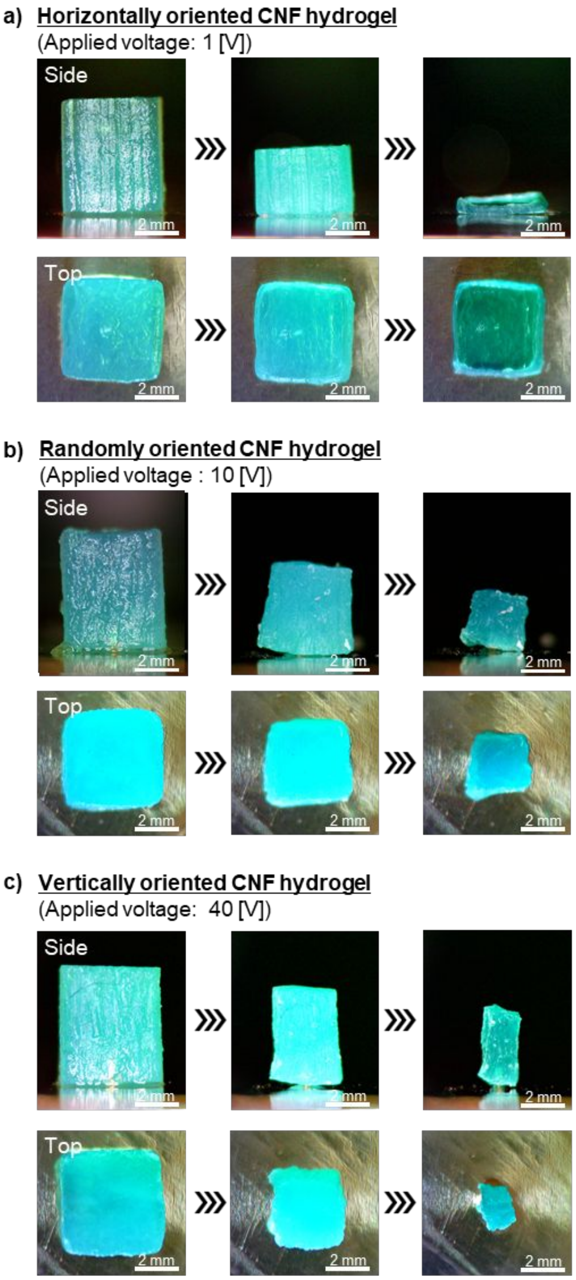

Figure S7. Drying-related shrinkage of CNF hydrogels. (a) Horizontally oriented CNF hydrogels with suppressed shrinkage in the horizontal direction. (b) Randomly oriented CNF hydrogels exhibiting isotropic drying and shrinkage to a certain extent. (c) Vertically oriented CNF hydrogels drying in the form of rods perpendicular to the anode.

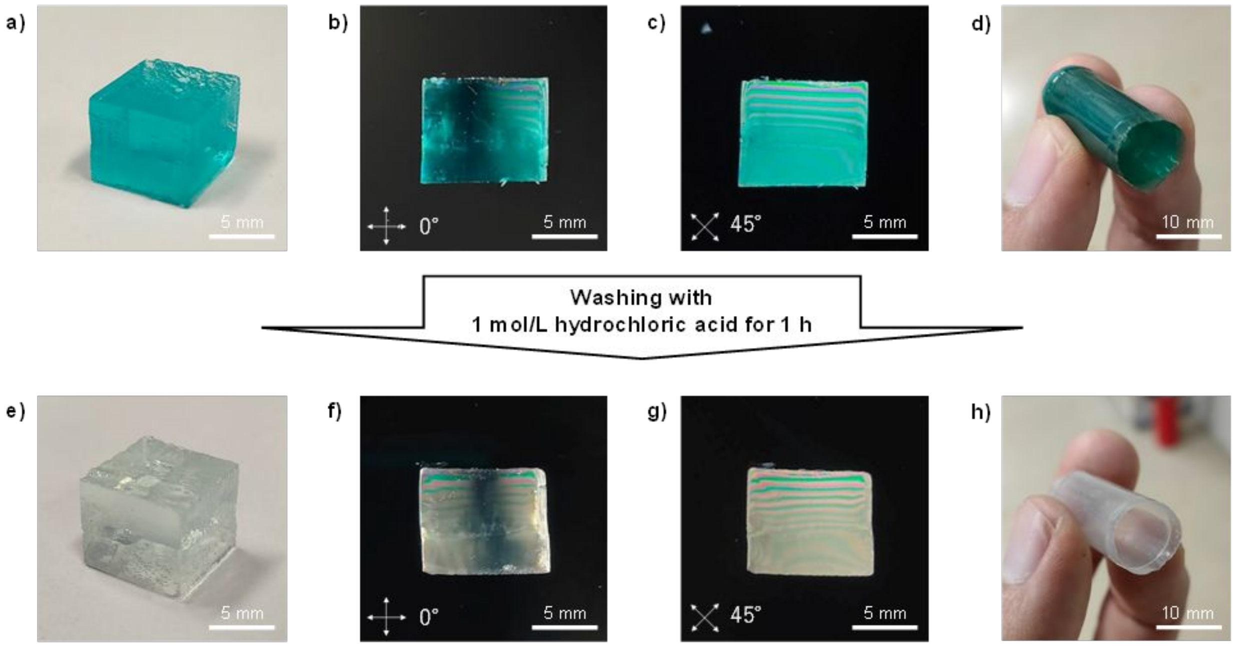

Figure S8. (a) CNF hydrogel prepared at an applied voltage of 1 V. (b), (c) Cross-section observed using a cross-polarizer. (d) CNF molding before washing. (e) CNF hydrogel after washing with 1 mol/L hydrochloric acid for 1 h. (f), (g) Cross-section observed using a cross-polarizer. (h) CNF molding after washing with 1 mol/L hydrochloric acid for 1 h.

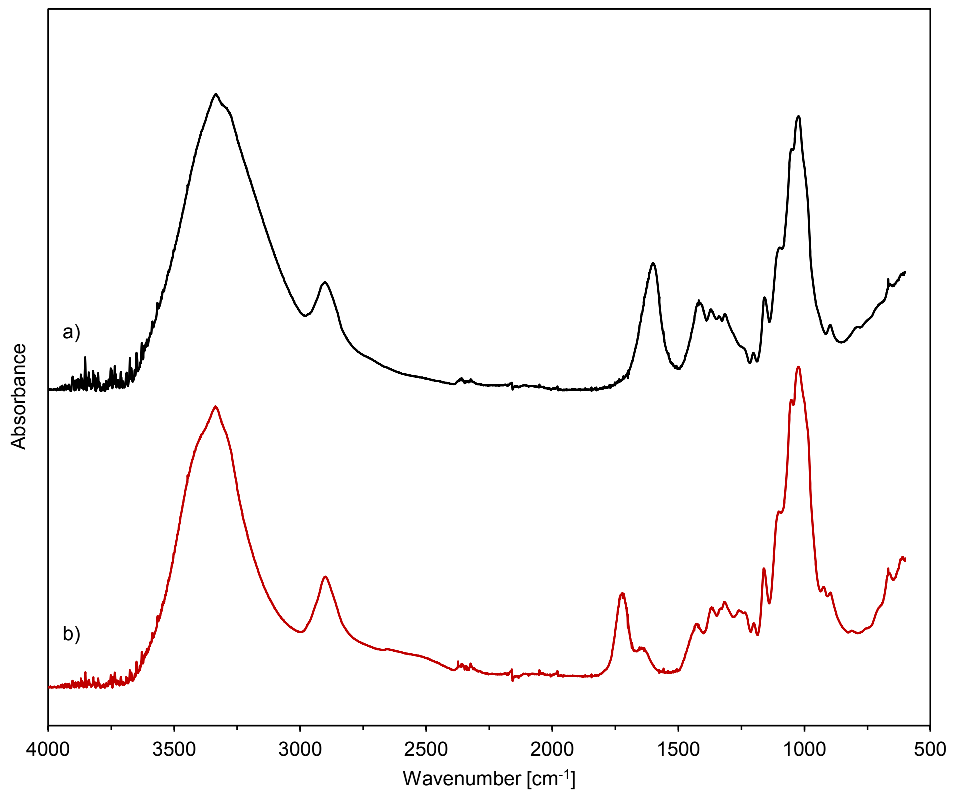

Figure S9. FT-IR spectra of CNF molding (a) before and (b) after washing with 1 mol/L hydrochloric acid for 1 h.

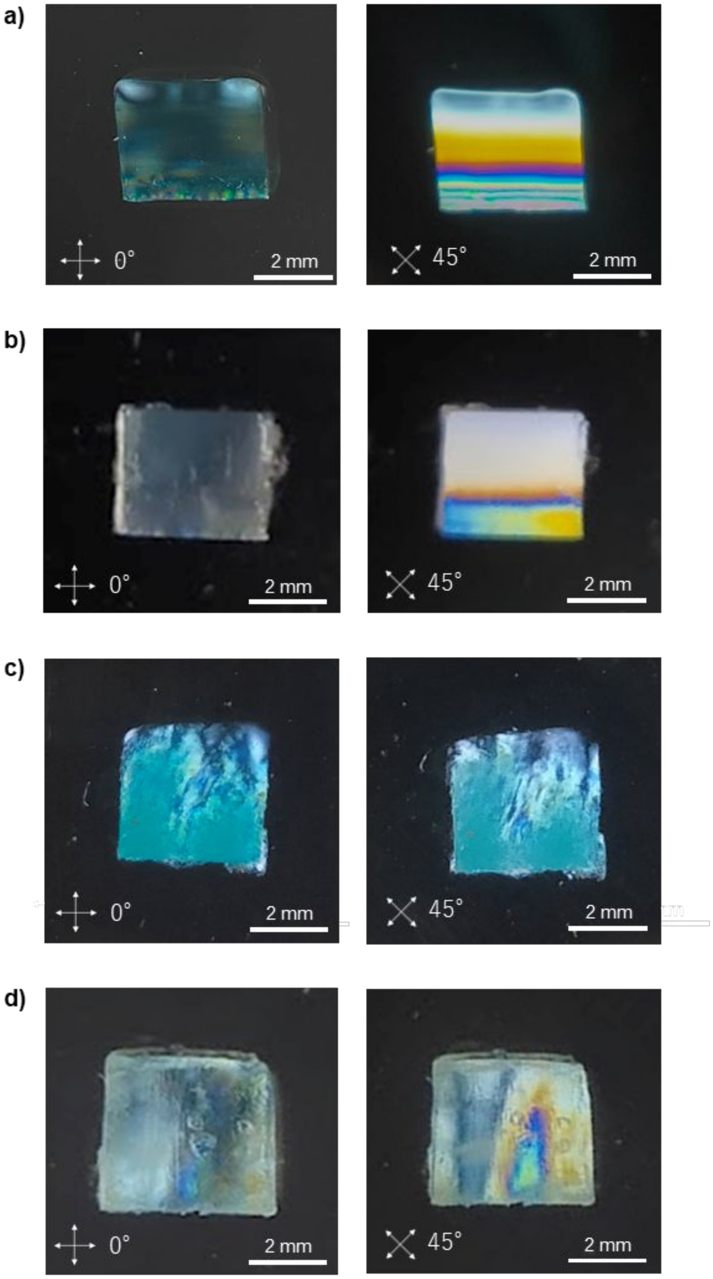

Figure S10. Cross-section of (a) an alginate hydrogel and (b) a nanoclay hydrogel prepared at applied voltage of 1 V observed through a cross-polarizer. Cross section of (c) an alginate hydrogel and (d) a nanoclay hydrogel prepared at 40 V observed through a cross-polarizer.

Table S1. Results of friction coefficient measurements of CNF hydrogels

	Static friction coefficient (μ_s)	Kinetic friction coefficient (μ_k)
Horizontally oriented CNF hydrogel	0.116	0.052
Randomly oriented CNF hydrogel	0.349	0.183

Table S2. Results of antiviral testing of molded CNF films against SARS-CoV-2

Virus	Film sample	log $TCID_{50}$/mL	LRV	Decrease rate [%]
SARS-CoV-2	CNF film_1	3.44	2.27	99.458
	PET film_1	5.70		
	CNF film_2	3.20	3.58	99.974
	PET film_2	6.78		
	CNF film_3	3.20	3.82	99.985
	PET film_3	7.02		

TCID50：median tissue culture infectious dose

LRV：Logarithmic Reduction Value

Supplementary methods

*Analysis***:** Fourier-transform infrared attenuated total reflection (FT-IR/ATR) spectra of the original CNFs and CNF aerogels were obtained using an FT-IR instrument (Frontier TN, PerkinElmer Inc., USA). The presence of sodium and copper ions was confirmed by energy-dispersive X-ray fluorescence (XRF; Shimadzu EDX-8000, Japan). The compressive moduli of the CNF hydrogels (8–9 wt%) on a 5 mm square cube were measured using a thermomechanical analyzer (TMA/SS6100; SII Nanotechnology Inc., Japan) equipped with a compression probe. The friction coefficient of each CNF hydrogel surface was measured using a tribometer (TYPE 14; Shinto Scientific Co. Ltd., Japan) at 300 mm/m and a load of 1 N. The randomly oriented CNF hydrogel, used for comparative analysis, was prepared using a previously reported procedure[S1] with a CNF concentration of 1 wt%. The drying-related shrinkage of the CNF hydrogel on a 5 mm square cube was monitored in a temperature and humidity chamber (PR-2 KT, ESPEC Corp., Japan) at 10 °C and 90% RH.

*Antiviral testing against SARS-CoV-2***:** Antiviral tests were outsourced to Chubu Food & Environmental Safety Center Co. Ltd. (Japan). Test samples were 30 × 30 mm, 100-µm-thick films prepared by drying the CNF hydrogel deposited on a flat copper plate at an applied voltage of 1 V. PET films were used as control samples (T60, Toray Industries, Inc., Japan), cut to the same size as that of sample. SARS-CoV-2 JPN/TY/WK-521 was used as the test virus, and VeroE6/TMPRSS2 cells (JCRB1819) were used as host cells. Dulbecco's modified Eagle's medium (Nacalai Tesque Co., Ltd., Japan) with 10% fetal bovine serum, penicillin (100 U/mL), streptomycin (100 µg/mL), and geneticin (G418) (1 mg/mL) was used as the cell growth medium. Dulbecco's modified Eagle's medium (Nacalai Tesque Co., Ltd.) with 2% fetal bovine serum, penicillin (100 U/mL), streptomycin (100 µg/mL), and geneticin (G418) (1 mg/mL) was used as the cell maintenance medium. Host cells were cultured in a monolayer in tissue culture

flasks using the cell growth medium. After monolayer culturing, the cell growth medium was removed from the flask and inoculated with the test virus. The cell maintenance medium was then added, and the cells were incubated in a CO_2 gas incubator (CO_2 concentration: 5%) at 37 °C ± 1°C for 5 d. The culture medium was then centrifuged at 3500 rpm for 10 min, and the resulting supernatant was used as the viral suspension. Samples were placed in sterile petri dishes and inoculated with 250 μL of virus suspension on the specimen surface and covered with glass to prevent sample wrinkling and drying. A cooling block (CoolBox XT refrigerated core, Azenta US, Inc., USA) was used to maintain the temperature at approximately 4 °C during incubation. Ten-times the amount of cell maintenance medium was added to the virus suspension after 2 h, the mixture was vigorously mixed again, and the sample was collected. The viral infection titer in the supernatant was determined. Testing was conducted in triplicate, with the control sample tested each time in a similar to the test sample, and the titer was measured after 2 h of action. Cell growth medium was used to culture cells in a monolayer in tissue culture microplates (flat-bottomed, 96-well). The medium was removed, and the cells were washed with cell maintenance medium. The supernatant of the mixture and the control were then diluted thrice with the cell maintenance medium, after which 100 μL of this diluted solution was inoculated into each well of the 4-well dish, and the cells were infected for 1 h in a CO_2 gas incubator (CO_2 concentration: 5%) at 37 ±1 °C. All stock solutions and diluents were removed after infection, washed twice with cell maintenance medium, and incubated in fresh cell maintenance medium for 3 d. After incubation, the cells were observed for morphological changes (cellular degeneration effect: CPE) using an inverted phase-contrast microscope and the 50% tissue culture infectious dose (TCID50) was calculated using the Behrens–Karber method and converted into virus infectious titer per milliliter of test solution.

References

S1. H. Dong, J. F. Snyder, K. S. Williams, J. W. Andzelm, *Biomacromolecules* **2013**, *14*, 3338–3345.
